# Response to the Toast, “History of Microbes”

**Published:** 1898-11

**Authors:** A. M. Cushing

**Affiliations:** Springfield, Mass.


### RESPONSE TO THE TOAST, "HISTORY OF MICROBES."

By Dr. A. M. Cushing, Springfield, Mass.,

At the Picnic of the Western Massachusetts Homœopathic Medical Society, at Forest Lake, Palmer, Mass., Sept. 14th, 1898.

*Mr. President, Ladies, and Gentlemen:* In coming before you upon this interesting but solemn occasion, I am reminded of the countryman's prayer, who said: O Lord, I am in trouble again. The last time I called on you was about five years ago, when a big bull was chasing me out of a pasture. Then I just *yelled* for help. I asked for breath and strength enought to get to the fence before the bull did, and I just did it; but the bull's horns perforated the posterior portion of my pantaloons. That was a pe-culiar predicament. I have been thankful ever since that I got to the fence before the bull did and that his horns were not any longer. Now I am in trouble again and want help. I am up a tree without a gun, and just in the edge of the woods is a bear and a catamount growling at each other; and I suppose they will get to fighting some time, and I fear it will not be till after dark, and I dare not get down till they begin to fight. I have been up this tree about three hours, and it is most night, and I want to go home, for the doctor says my baby has got the appendicitis in its throat, and I suppose my wife is out with a broom looking for me. All I ask is that those animals may get to fighting pretty soon, and I will get down and run for home and ask no more favors."

Now, Mr. President, I am up a tree without any gun. If some one will get up a little excitement outside, I will down and start for home, and be thankful if I only get to the depot; if not—I must give you a history of microbes!

In the forty minutes alloted me you must not expect me to give you a microscopic description of all the varieties of microbes. When microbes first came on the market I did not believe in them. I thought they were a hum-*bug* instead of a worm; but when I found so many people interested in them, I decided to investigate, and found there were a great many—large and small. There are some so small we have not found them yet. We have not got a thermometer that goes high enough. I have been looking for one kind several years, but have not found it. I think I will have to get a gold-bowed microscope. When I found how many microbes there are, I decided to make a microbe-killer. I thought it would be a great professional and financial success; but before I got it finished, I concluded if the Almighty wanted to kill a man, he would take something larger than a microbe, as he had a plenty of larger things. Then I thought that possibly those little things were not made to kill us, but were for our good. As I could find no one who knew anything certain about it; right in the face of a big Klondike fortune (which is usually a failure or death), I abandoned the whole thing—a great loss, no doubt, to the world and to me.

It is wonderful how many microbes there are. I doubt if a majority of people mistrust how many microbes there are. They are everywhere. When they get to running around in a man's head, they say he has wheels on his brain. If they get into his flesh, they say he has rheumatism. If they get into his feet, they say he has gout. If they get to sitting on his toes, they say he has corns. If they crawl into a hollow tooth, they halloo for a dentist. If they get to singing psalms in one's ears, they think they have been taking Quinine. If a stray one gets into the eye, they say they have strabismus. If they get to telegraphing on the facial nerve, they say they have "*tic*-dolly-roo." If they get to playing lawn tennis in the nose, they say we have hay-fever. If they get to dancing on the tongue, it raises mischief in the neighborhood, for the tongue can't be kept still. If it is a man's tongue, it kills him, sure. If they get to raising young ones in the throat, they say we have diphtheria. If they try to stop the heart beating, they say we have "angelina petroleum." If one gets crosswise in a bronchial tube, they call it bronchitis or pneumonia. If they get packed into the liver, they say we are bilious. If they get to raising the wind—in the stomach, they say we have gas, or gastritis, or gastralagia, or gastrodominoes, or dyspepsia. If they get to running bicycle races in the bowels, they say we have diarrhœa; if there is a bad accident, they call it dysentery.

We have had these microbes a long time; ever since Adam ate that first wormy apple. More than *fifty years ago* I saw microbes crawl right out of old cheese, and hop and jump and "skipper" around the table. When I was a little boy—I never was a little girl, for my mother would not be starved nor stall-fed for the sake of having a little girl, she did not believe in that nonsense and I was glad of it; so I was a little boy. When I was a little boy, children had microbes. I had them, sometimes five or six inches long, and had to take "Pink and Seeney." My mother had microbes; she had eight. I was one of them. That is all I know about microbes.

---

THE medical officer to the Moss Side District Council (Manchester) has called special attention to the high mortality from summer diarrhœa. The prolonged high temperature has no doubt, he says, had a great influence in producing the disease. High temperature of the air has been proved, however, to bear only an indirect relationship to its occurrence. Pollution of the soil by dead organic matter, neglect of the removal of dry refuse, and, as the result, the pollution of the air we breathe and of food, are the most essential and most prevalent factors in producing summer diarrhœa. This cannot be too strongly impressed upon householders, and especially upon those who have the care of young children.—*Health* for October.

